# What Kigali’s open-air markets reveal about achieving food and nutrition security: the role of African indigenous crops

**DOI:** 10.1186/s40066-022-00359-4

**Published:** 2022-02-14

**Authors:** Eugene Baraka, Mary S. Willis, Brice A. Ishimwe

**Affiliations:** 1 Department of Epidemiology, Biostatistics and Occupational Health, McGill University, Montréal, Québec H3A 1A2 Canada; 2 Department of Nutrition and Health Sciences, University of Nebraska-Lincoln, Lincoln, NE 68583 USA; 3 Department of Environmental Studies, University of Lay Adventists of Kigali, Kigali, Rwanda

**Keywords:** Rwanda, Dietary diversity, Food security and nutrition security, Indigenous species, Malnutrition, Open-air markets

## Abstract

**Background:**

Household dietary diversity in Rwanda remains low and significantly contributes to the double burden of malnutrition. Rwanda has one of the highest under five stunting rates globally, and malnutrition remains one of the most pressing public health issues; therefore, factors that shape food and nutrition security are of utmost concern. Globally, the variety of foods available in open-air markets has been shown to affect dietary diversity. Furthermore, the consumption of indigenous foods can contribute to a diverse diet and improve nutrition status. At present, there are limited data on foods available for purchase in open-air markets in Africa. Therefore, this study was designed to provide data on food availability in the largest open-air markets of Rwanda’s most populated city, Kigali, and to highlight which foods indigenous to Africa can be purchased.

**Methods:**

All consumables were inventoried between October and December of 2020 in three open-air markets of Kigali, the capital city of Rwanda. Consumables were organized by the site of domestication and the nutritional contents of some African indigenous crops were compared to similar non-indigenous items.

**Results:**

A variety of raw and processed consumables was available in the open-air markets inventoried; however, only 25.8% of available species are indigenous to Africa. All Rwanda’s staples, including sweet potatoes, plantains, beans, maize, banana, and cassava, are endemic to other continents. Indigenous plant species, which are often drought-resistant and more nutritious, for example, Africa’s pineapple fruits (*Myrianthus holstii*), could not be purchased in Kigali’s open-air markets. Pineapple fruits are richer in iron, vitamin C, protein, and vitamin A than banana, which is the most consumed fruit in Rwanda.

**Conclusions:**

Given rapid population growth, limited arable land, and erratic climate patterns, policies to conserve and promote indigenous species, especially those already adapted to harsh environmental conditions, should be enacted in Rwanda. The cultivation of native vegetables and fruits in home gardens, and the conservation of edible wild species, can improve dietary diversity and enhance food and nutrition security across the entire country.

## Background

Food availability, along with accessibility, useability, and stability and/or sovereignty, are central to the concept of food and nutrition security [1]. Yet in many countries, assessments of how these food security pillars function to impact household health and wellbeing have not been made [1]. Within each country and region, the food system is complex and affected by a plethora of issues. For example, in Rwanda, food availability has been shaped by factors such as geography, climate, population growth, colonization, farming practices, and government policies. Ninety percent of cropland is sloped, arable land is limited, and climate is conditioned by this terrain [2]. Given these conditions, climate change will worsen an already precarious agricultural production system and increase food and nutrition insecurity [3]. Rwanda is the second-most densely populated country in Africa, with an annual population growth rate of 3 to 3.1%, thus even more land is needed to accommodate this accelerated growth [4]. Historically, colonization by Germany and Belgium brought about the holistic destruction of the traditional food system, whereby foods indigenous to the Americas, Europe, and Asia were prioritized [5]. For example, cultivation of cash crops was mandated by the Belgians and, gradually, traditional farming systems with intercropping have been replaced with high-yielding production methods [6]. Finally, government policies such as the Crop Intensification Program (CIP) have prioritized increased productivity of just eight crops, i.e., maize, rice, wheat, beans, soybean, cassava, Irish potato, and banana [2, 7]. None of these crops are indigenous to Africa, but more importantly, they are not well positioned to address micronutrient malnutrition, improve food and nutrition security, or increase household dietary diversity [8].

Over the past 2 decades, Rwanda has made substantial economic progress which has improved the standard of living throughout the country [8]. Under five stunting has decreased, from 44.3% in 2010–11 to 33% in 2019–20, while child obesity rates have dropped from 6.0% in 2012 to 5.6% in 2019 [9, 10]. Despite this progress, the rates of malnutrition, especially under five stunting, remain unacceptably high [8]. In addition, adult obesity has escalated in the past decade and anemia, caused by iron deficiency, remains one of the most pressing micronutrient related diseases [3, 11]. Furthermore, the introduction of a Western-style diet worldwide, including in Rwanda, as well as the nutrition transition, are increasing nutrient deficiencies such as protein, vitamin A, and zinc [11, 12].

African indigenous food crops play an important role in the wellbeing of people in many countries and regions of the continent [13]. First, they are usually more affordable than alien crops [14] and many are richer in macro and micronutrients compared to their exotic counterparts [15, 16]. They are also better able to withstand pests and diseases, and are more tolerant to drought, heat, and low fertility soils, i.e., they are well adapted to the environment in which they originated [17]. Nonetheless, as in many other regions of the world, high-yielding species such as maize and rice continue to be prioritized over indigenous food crops, with monocropping replacing intercropping [18]. Cash crops for export, such as tea, now occupy the lands that would otherwise be important for cultivating diverse food crops, including indigenous ones, and create a greater dependence on foreign nations for the import of subsistence foods [19]. Because approximately 66.7% of Rwandans are under the age of 25, the knowledge of indigenous plants and animals, as well as the importance of consuming a diverse diet, fades with each generation [20]. Ultimately, the knowledge of Africa’s endemic species, and their cultivation and conservation, as well the nutritional and medicinal value, may be completely lost [20].

Access to a diversified diet has been correlated with improvement in household nutrient intake and reduction of under-five stunting [21]. Moreover, dietary diversity has been positively associated with a greater variety of consumables available for purchase in local markets [22]. Although there have been a variety of food security assessments conducted in Rwanda, to our knowledge, none of them have focused on food availability, or generated inventories of plant and animal species for sale, in large open-air markets in any urban or rural setting in Rwanda. Because of the agricultural priorities for production, and the high rates of malnutrition that exist, investigation of what can be procured from Rwanda’s markets was initiated. Given the contributions that indigenous foods make to achieving dietary diversity and improving human health, and their adaptation to local environmental conditions, it is important to know which, if any, of these are available as well. Thus, the aim of this study was to begin documenting crop and livestock availability, starting with open-air markets in the largest city, Kigali, to identify foods that could be purchased, as well as their continent of origin, and illuminate the proportion of cultivated or wild foods native to Africa. From this data, we make recommendations to increase crop diversity and eliminate or mitigate the impact of macro and micronutrient deficiencies. Finally, the contribution that African indigenous crops can make in improving food and nutrition security in Rwanda is highlighted.

## Materials and methods

### Study setting

Rwanda (1.9403 °S, 29.8739 °E) is a 26,338 sq. km land-locked country in East Africa with a population of approximately 12.6 million [10]. About 70% of the Rwandan population practices agriculture and nearly 72% of the workforce is employed within the sector [9]. As of 2019, agriculture accounted for up to 33% of the total national GDP, where tea and coffee are the main exported crops, in addition to dry beans, potatoes, rice, cassava, and maize [9]. Rwanda is divided into five provinces, namely Northern, Southern, Eastern, Western, and Kigali, with the latter being both province and capital city in the geographic center of the country.

**Fig. 1:**
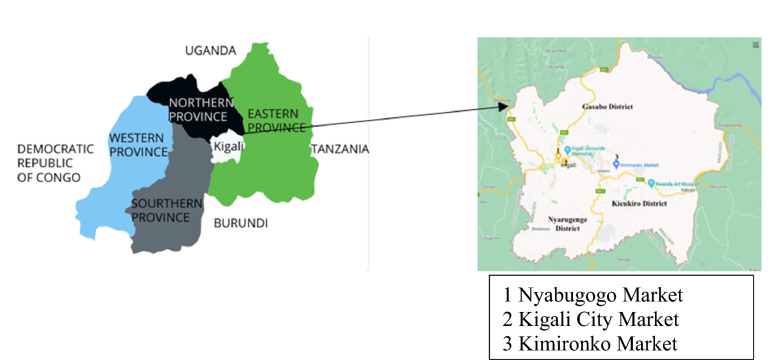
Map of Rwanda (left) and Kigali with the sampled open-air markets (right)

Because there are no published data for any of Rwanda’s provinces, three open-air markets in the largest and most populated city, Kigali (1.9441 °S, 30.0619 °E), were selected as initiatory study sites; *Nyabugogo* Market, *Kimironko* Market, and Kigali City Market (Fig. 1). *Nyabugogo* is the site for all of Kigali’s market transactions and pricing, and both *Nyabugogo* and *Kimironko* are among the city’s largest open-air sites for purchasing consumables [23]. Kigali City Market represents the latest trend in food shopping, combining an expansive open-air market within a shopping mall. Because open-air markets allow shoppers to bargain, and offer the largest number of raw rather than processed foods, they remain an optimal site for obtaining ingredients for food preparation. In contrast, kiosks in Rwanda obtain their foods from *Nyabugogo* Market [23] and supermarkets sell foods that are more processed at a higher price; hence, open-air markets are preferable for procuring the main agricultural products and assessing available foods in Kigali.

### Data collection

All data from the three markets were collected between October and December of 2020. Data collection consisted of walking through each market to record all consumables for sale, and subsequently, the inventories for each open-air market were combined in MS excel, where items were organized in tables by food category and continent of domestication. Categorization of consumables was made based on the *FAO/WHO Individual Food Consumption methodology for groups and subgroups* [24]. To ensure that the widest number of consumables were recorded, all markets were visited twice. Also, before visiting the markets, the peak period for visitation during a day was determined.

### Data analysis

After combining all the inventories from the markets by their food category, their place of origin was identified. Food products that are indigenous to Africa, or to other continents, were verified using previously published articles, the three-volume series of *Lost Crops of Africa*, and platforms such as WFP, WHO, and FAO. Four separate tables were made for consumables indigenous to world regions, including Africa, the Americas (North, Central, and South), Asia, and Europe (including the Mediterranean). For each food item, an English, scientific, and local (*Kinyarwanda*) name was identified, where possible. Total numbers of foods indigenous to each continent were counted and the nutritional values of African consumables were compared to similar items from other continents. If different parts of a food item or crop were found in the markets, the item was counted once in the final species’ tally. For example, if both cow’s milk and beef were inventoried, the species (*Bos taurus*) was counted one time to represent both items.

## Results

A total of 62 species of plants or livestock from 4 different continents, including Africa, the Americas, Asia, and Europe/Mediterranean, were found within the three open-air markets in Kigali (Table 1). These species fall within seven of the FAO/WHO food categories: cereals, fish, fruits, legumes, root crops, meat/dairy, and vegetables (Table 1). Fruits, followed by vegetables, included the largest number of species, 32% and 18%, respectively (Table 1).

**Table 1:** FAO/WHO food categories by number and percent in Kigali Markets

Category	Number of species	Percent (%)
Cereals	6	10
Fish	10	16
Fruits	20	32
Legumes	3	5
Root crops	6	10
Meat/dairy	6	10
Vegetables	11	18
Total	62	100

### Indigenous foods of Africa

Sixteen of 62 total species found in the markets are indigenous to Africa and fall within five FAO/WHO categories: cereals (*n* = 2), fish (*n* = 8), fruits (*n* = 2), meat/dairy (*n* = 2), and vegetables (*n* = 2). Cereals indigenous to Africa, and found within the open-air markets, are pearl millet and sorghum; however, they were only sold in flour form. Millet flour was found in two of the three markets inventoried, while sorghum flour was present in each market. All three open-air markets were abundantly supplied with scores of fish species endemic to Africa and both dried and fresh forms were available. Small fish species, especially *indagara* and silver *cyprinid*, were generally more plentiful than larger fish species. Although the markets had an abundance of fruits, only watermelon and African eggplant are of African origin. Both rabbit (*Lepus spp.)* and cow (*Bos taurus africanus)* from the meat/dairy category were sold, but only in fresh meat form. Cow’s milk was also sold in each of the markets. Additionally, mushrooms and lettuce were the only vegetables indigenous to Africa that could be purchased in Kigali’s markets. All vegetables were sold fresh and had been grown on local farms (Table 2).

**Table 2:** Food indigenous to Africa

Common name	Scientific name	Local name
Cereals		
Pearl millet	*Pennisetum glaucum*	*Uburo*
Sorghum^a^	*Sorghum bicolor*	*Amasaka*
Fish		
*Kapenta*	*Limnothrissa miodon*	*Indagara*
Nile tilapia	*Oreochromis niloticus*	
African catfish	*Clarias gariepinus*	
*Haplos*	*Haplochromis sp.*	
Silver cyprinid	*Rastrineobola argentea*	
Marbled lungfish	*Protopterus aethiopicus*	
*Ningu*	*Labeo victorianus*	
Tanganyika killifish	*Lamprichthys tanganicanus*	
Fruits		
African eggplant	*Solanum macrocarpon*	*Ikiringanya*
Watermelon	*Citrullus lanatus*	Wotameloni
Meat/dairy		
Sanga cattle (African cattle)	*Bos taurus africanus*	*Inka*
Rabbit	*Lepus spp.*	*Urukwavu*
Vegetables		
Lettuce	*Lactuca sativa*	
Mushrooms	*Termitomyces microcarpus*	*Imegeri*

^a^Staple food

### Indigenous foods of the Americas

Seventeen species inventoried in the markets are indigenous to the Americas (Table 3). Foods from the Americas fall within five categories: cereals (*n* = 1), fruits (*n* = 9), legumes (*n* = 2), root crops (*n* = 3), and vegetables (*n* = 2). The most important staples of Rwanda, maize, sweet potatoes, and cassava, originate in the Americas and were found in each of the three markets inventoried. In the cereals’ category, maize could be purchased as either grain or flour. Maize grain was either dried or fresh. Dried maize is sold without the cob, and was harvested and dried in previous agricultural seasons. Fresh maize is sold on the cob and is purchased to be roasted or steamed. All the markets had a variety of fruits from the Americas; in fact, the largest number of fruits in all open-air markets were from both this continental landmass and Asia, and were all sold in a fresh, ready-to-eat form. Beans and groundnuts were the legumes from the Americas that were available in all the open-air markets and all were sold fresh as well. Cassava flour, used to make a typical Rwandan dish, *ugali,* could also be purchased. Finally, green beans, cassava leaves, amaranth, and *chayote* were the vegetables native to the Americas in Kigali’s open-air markets (Table 3).

**Table 3:** Food indigenous to the Americas (North, Central, and South)

English name	Scientific name	Local name
Cereals		
Maize^a^	*Zea mays*	*Ibigori*
Fruits		
Avocado	*Persea americana*	*Avoka*
Guava	*Psidium guajava*	*Amapera*
Passion fruit	*Passiflora edulis*	*Marakuja/itunda*
Pineapple	*Ananas comosus*	*Inanasi*
Pumpkin/Zucchini	*Cucurbita spp.*	*Igihaza*
Pepper (bell, chili)	*Capsicum spp.*	*Puwavuro*
Strawberries	*Fragaria* × *ananassa*	*Inkeri*
Tamarillo/tree tomato	*Solanum betaceum*	*Ikinyomoro*
Tomato	*Solanum lycopersicum*	*Inyanya*
Legumes		
Beans^a^	*Phaseolus spp.*	*Ibishyimbo*
Peanuts^a^	*Arachis hypogaea*	*Ubunyobwa*
Root crops		
Cassava^a^	*Manihot esculenta*	*Imyumbati*
Potato^a^	*Solanum tuberosum*	*Ibirayi*
Sweet potato^a^	*Ipomoea batatas*	*Ibijumba*
Vegetables		
Green beans	*Phaseolus vulgaris*	*Imiteja*
Cassava leaves	*Manihot esculenta*	*Isombe*
Amaranth	*Amaranthus spp.*	*Imboga rwatsi*
Chayote	*Sechium edule*	*Ibishayoti/Ibidodoyi*

^a^Staple food

### Indigenous foods of Asia

Consumables endemic to Asia equaled 16 species, including cereals (*n* = 2), fruits (*n* = 9), meat/dairy (*n* = 1), root crops (*n* = 1), and vegetables (*n* = 3) (Table 4). The foods from Asia include those native to Eastern, Southern, and South-Eastern Asia. Rice and sugarcane were the cereals of Asia that were available for sale in the Kigali open-air markets. Raw sugarcane stalks were sold in one of the three markets, while processed sugarcane in the form of granulated sugar was available in all of the markets. Plantains were widely available, along with other fruits such as avocado and tomato, and all were sold fresh from local farms. Chicken meat, in addition to eggs, could also be purchased in all inventoried markets. Finally, fresh vegetables including onions, leek, and garlic were available for sale within all of the open-air markets inventoried (Table 4).

**Table 4:** Food indigenous to Asia

English name	Scientific name	Local name
Cereals		
Rice^a^	*Oryza sativa*	*Umuceri*
Sugarcane	*Saccharum officinarum*	*Igisheke*
Fruits		
Apple	*Malus pumila*	*Pome*
Banana	*Musa acuminate*	*Umuneke*
Cucumber	*Cucumis sativus*	*Cocombre*
Eggplant	*Solanum melongena*	*Intoryi*
Lemons	*Citrus limon*	*Indimu*
Mandarin orange	*C. reticulata*	*Icunga*
Mango	*Mangifera indica*	*Umwembe*
Papaya	*Carica papaya*	*Ipapayi*
Plantains^a^	*Musa* × *paradisiaca*	*Igitoki*
Legumes		
Soybeans	*Glycine max*	*Soya*
Meat/dairy		
Chicken	*Gallus gallus domesticus*	*Inkoko*
Root crops		
Taro	*Colocasia esculenta*	*Amateke*
Vegetables		
Onions		*Igitunguru*
Leek		
Garlic	*A. sativum*	*Tungurusumu*
Miscellaneous		
Eggs	*Gallus gallus domesticus*	*Amagi*

^a^Staple food

### Indigenous foods of Europe

European indigenous foods included those native to Europe and the Mediterranean regions, covering Western and Southern Europe, North Africa, and Western Asia. Thirteen total species indigenous to Europe/Mediterranean were found in the markets inventoried (Table 5). These are categorized in six different groups, including cereals (*n* = 1), fish (*n* = 2), legumes (*n* = 1), meat/dairy (*n* = 3), root crops (*n* = 2), and vegetables (*n* = 4) (Table 5). Meat of both cow, goat, and pig were available in each of the markets, as was cow’s milk. Wheat was sold in flour form and in a variety of wheat-based processed foods, including noodles, *samosas*, fritters, and bread (Table 5).

**Table 5:** Food indigenous to Europe/Mediterranean

English name	Scientific name	Local name
Cereals		
Wheat^a^	*Triticum aestivum*	*Ingano*
Fish		
Common carp	*Cyprinus carpio*	
Sardines	*Sardina pilchardus*	*Saladine*
Legumes		
Peas	*Pisum sativum*	*Amashaza*
Meat/dairy		
Cow	*Bos taurus*	*Inka*
Cow’s milk	*Bos taurus*	*Amata*
Goat	*Capra hircus*	*Ihene*
Pork	*Sus scrofa domesticus*	Ingurube
Root crops		
Carrots	*Daucus carrota*	*Karoti*
Beets	*Beta vulgaris*	*Beterave*
Vegetables		
Celery	*Apium graveolens*	*Seleri*
Cauliflower	*Brassica oleracea var. botrytis*	
Cabbage	*B. oleracea var. capitata*	*Amashu*
Chard	*Beta vulgaris*	

^a^Staple food

**Fig. 2:**
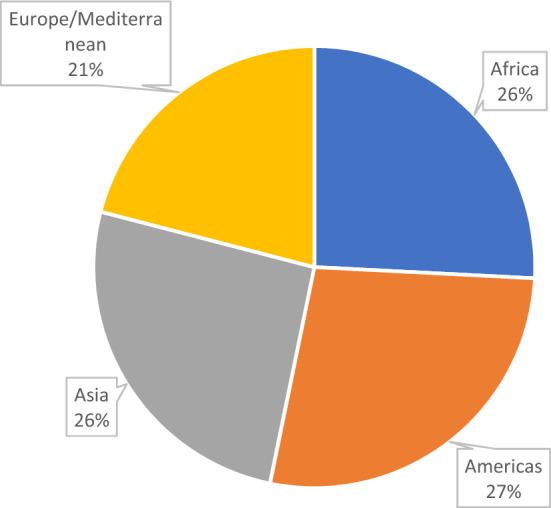
Species indigenous to each continent by Percent

Overall, just 25.8% or 1/4th of the species sold in the sampled Kigali markets are indigenous to Africa, while the remaining 74.2% are exotic species from either the Americas, Asia, or Europe (Fig. 2). Similar percentages of the available crops for sale in Rwandan markets had originated in Asia and the Americas, while Europe/Mediterranean was the origin for the smallest percentage of foods available in Kigali’s markets at 21% (Fig. 2).

## Discussion

If the world is to achieve food and nutrition security, policies need to promote agricultural productivity, while also emphasizing nutrition-sensitive agriculture that increases dietary diversity [25]. Recent research has attributed the rise of obesity and slow reduction of undernutrition to the decline in dietary diversity among the world’s population [26–28]. This decline in the consumption of a diverse diet is mostly due to globalization, and the trending worldwide nutrition transition, as both of these factors lead to the neglect of wild and indigenous foods and their cultivation, as well as the loss of knowledge for their preparation and consumption [20]. In light of the many factors that have shaped Rwanda’s food system, we inventoried consumables in three of Kigali’s open-air markets to illuminate food availability.

### Cereals

Cereal crops for sale in the sampled markets included pearl millet and sorghum native to Africa, maize indigenous to the Americas, rice and sugarcane indigenous to Asia, and wheat from Europe/Mediterranean (Tables 2, 3, 4, 5). Finger millet (*Eleusine coracana*), a variety of African indigenous millet species, was not present in any of the markets inventoried. Native sorghum and millet have been reliable foods in Rwanda for millennia, long before Europe’s contact with Africa, as shown by recent archaeobotanical studies [29]. Finger millet was used in various spheres of nutrition, such as making porridge and traditional wine brewing [30]. As one of the most nutritious among cereals, finger millet is rich in methionine, a critical amino acid lacking in many diets worldwide [18]. Another important trait of finger millet has to do with seed longevity; they can be stored and remain viable for longer periods of time compared to other grains [18]. Furthermore, finger millet is the most drought and heat resistant of the major cereals including rice, wheat, and maize [18].

In comparison to maize, pearl millet’s iron content is three times higher (9.8 mg per 100 g), while the protein content is higher too (11.8 g per 100 g) [18]. Although somewhat like maize, sorghum has higher levels of B vitamins (pantothenic acid, niacin, folate, and biotin) and generally higher protein content, depending on the variety [18]. Its drought-tolerant ability allows it to grow well under harsh conditions, an environment that neither wheat nor maize can survive [18, 31]. Sorghum is also more effective for absorbing and maintaining water compared to maize [32] and its cuticle adds extra resistance against flooding and pests [33]. Like pearl millet, sorghum is also at high risk of extinction in Rwanda [20].

Despite their nutritional potential, the cultivation of sorghum and millet has been significantly reduced over the past years in Rwanda for a variety of reasons, including loss of knowledge about traditional farming practices [34] and agricultural policies that focus more on high productivity and less on nutrition [8]. Although exotic cereals serve as great nutritional complements to both millet and sorghum, they are rapidly replacing the once-cherished indigenous cereals [20]. Also, more farmers are abandoning indigenous millet and sorghum for maize and, in some cases, cultivating maize in areas where it is poorly suited and cannot consistently perform well [18].

### Fish, meat, and dairy

Fish farming began in Rwanda in late 1940s during the Belgian colonial rule and has since greatly contributed to Rwandan household’s food security [35]. Well over 5% of Rwanda’s total area is made of water bodies and the majority of fishing is done in Lake Kivu [36]. Most fish species found in the markets are indigenous to Africa and this is the category with the highest number of indigenous species by percentage (Tables 2, 3, 4). Rwanda is by far one of the largest importers of fish in East Africa, buying from countries like Uganda, Tanzania, and Burundi, not to mention the imports from China and other European countries [36, 37]. Despite importing large amounts of fish, per capita fish consumption in Rwanda is only 2.3 kg per year, the lowest in East Africa (where the average consumption is 6.7 kg per annum), but also far below WHO’s recommendation of 14.9 kg per capita annually [38]. Ironically, fish are imported and subsequently exported to the Democratic Republic of Congo [35, 37]. According to the available literature, there is no known difference between the nutritional contents of African indigenous and non-indigenous fish species. Nevertheless, by maximizing the use of natural resources and endemic fish species, Rwanda could improve fish production, increase biodiversity, lower the cost, and increase consumption [39].

Cattle is the most important livestock in Rwanda; it is the only source of milk and the largest provider of meat [40]. The most dominant cattle type is *Ankole* in the *Sanga* cattle category, a species indigenous to Africa and primarily used for milk production [40]. Because the indigenous *Ankole* breed is not a top meat producer, the Rwandan Government has recently introduced the *Fleckvieh* breed that serves a dual purpose, producing both milk and meat, and the *Friesian* breed that is a great milk producer [40, 41]. As the per capita meat and milk consumptions in Rwanda are substantively below the FAO/WHO’s recommendation in Rwanda [42], the introduction of these exotic breeds has the potential to increase both milk and meat production, hence reducing malnutrition. Although these non-indigenous cattle breeds are greater producers compared to their indigenous counterparts of Africa [43], the latter are better adapted to the environment, able to tolerate significant harsh environmental conditions, have higher disease tolerance, and can utilize limited forage [44, 45]. Therefore, leveraging the indigenous species’ ability to thrive, along with making the most out of the greater milk and meat producers, could significantly not only improve availability, but also conserve biodiversity and prevent the loss of these indigenous species.

While cattle are the most common livestock in Rwanda, they have long reproductive cycles and are hard to raise for farmers with small land holdings. It may take over 10 years to double a herd of 100 cattle, while a herd of 100 indigenous sheep and goats can be doubled in only 4 and 6 years, respectively [46]. Therefore, focusing on diversifying livestock meat production by rearing small indigenous ruminants such as goat and sheep, and producing more chickens for meat and eggs, would potentially bring about a decrease in protein deficiency and alleviate poverty, especially among small-holder farmers [46].

### Fruits and vegetables

Out of 18 total species, African eggplant and watermelon are the only fruits indigenous to Africa found in the Kigali open-air markets (Table 2). The majority of Rwandans rely on banana, a fruit indigenous to Asia, as a main source of carbohydrates; and Rwanda is the second largest banana consumer worldwide, with an annual per capita consumption of approximately 144 kg [47]. However, most of the available banana cultivars are low-yielding and susceptible to pests and diseases [48] and are, therefore, not a sustainable solution to reducing malnutrition in Rwanda. Although indigenous fruits often play an important role in household food security, and are used to make products ranging from medicine to oil [42], they rarely make it to the open-air market.

One of the most micronutrient-rich indigenous fruits of Africa, the African pineapple-fruits (*Myrianthus holstii*), was not found in any of the Kigali markets. However, in comparison to banana, African pineapple fruits have a higher iron content (16.3 mg versus 0.3 mg/100 g), greater amounts of vitamin C (19.80 mg versus 8.7 mg/100 g), higher protein (8.03 g versus 1.1 g/100 g), higher vitamin A (0.933 mg versus 8.2 mcg/100 g), and higher zinc levels (2.327 mg versus 0.15 mg/100 g) [49, 50]. The paucity of indigenous African fruits in markets implies that access is limited and/or that there is a decline in the consumption and/or cultivation of these nutritious species.

Vegetables indigenous to Africa, and found in the Kigali markets, included lettuce and mushroom species, representing only 14% of all vegetables for sale. Vegetables in the open-air markets were dominated by onions, cabbages, and cassava leaves (*isombe*). Cassava leaves have become a staple for Rwandans and constitute a substantial part of a household’s daily intake [43]. However, in comparison to cassava leaves, cowpea leaves are protein (21.37 g versus 5.5 g/100 g), iron (13.89 mg versus 4.2 mg/100 g), and zinc (5.22 mg versus 0.01 mg/100 g) and each of these nutrients is deficient in the diets of Rwandans [10, 51, 52].

### Legumes

Rwanda is the highest per capita bean consumer worldwide, with 35 kg per person per year [25]. Beans are the most consumed legume in the country and are the source of protein intake for families that cannot afford meat [53]. Additionally, the current bean varieties have been biofortified with iron to reduce the rate of anemia; however, only 20% of beans are biofortified and just 15% of the population have access to iron-fortified varieties [53, 54]. Fortification of crops can allow consumers to reach the recommended levels of micronutrients, but biofortified seeds are more expensive and require the purchase of inputs. This typically means that they are less accessible to rural consumers [55]. Therefore, biofortification may not be a sustainable method for reducing micronutrient malnutrition, especially if the biofortified seeds and monocropping are applied without inclusion of indigenous species and traditional farming. Promoting biodiversity by including indigenous legume species, for example cowpea (*Vigna unguiculate*) and pigeon pea (*Cajanus cajan*), in the diets of Rwandans can reduce costs, increase accessibility, increase sustainability, and provide the required micronutrients for those who are deficient.

## Limitations

This study has several limitations. The first and most important limitation is that the open-air markets were inventoried during the COVID-19 pandemic. This could have prevented some sellers from bringing food to the markets, due to isolation or concern about contracting the virus. As a result, we could have missed some of the most important indigenous or alien crops for sale, ultimately affecting the overall count of available species. In addition, the research was only conducted in the capital city of Kigali, but rural areas are known to consume more indigenous foods than urban centers; therefore, this study is by no means meant to be generalizable to all provinces in Rwanda. Even so, Kigali is in the heart of Rwanda and receives produce from around the country to support the capital city’s large population. Lastly, some indigenous foods are seasonal and may have not been available in the markets at the time of our data collection. Although the data generated by this first inventory of Kigali’s largest markets can only represent a snapshot of available foods, including those which are indigenous to Africa, it provides a starting point for future research. Additional research to determine the availability of consumables in rural areas and the other provinces around Kigali Province, is needed to gain a comprehensive understanding of the country’s production of indigenous foods and access to wild species.

## Conclusion and recommendations

The purpose of this study was to begin documenting available foods in open-air markets, starting with the largest open-air sites in Rwanda’s capital city, Kigali. Using the inventory, we identified the continent of origin for each food, and illuminated the proportion of cultivated or wild foods native to Africa which were available in the sampled markets. As Rwanda repositions itself globally, expanding its collaboration with other countries, and attracting investors from around the world, adoption of other foodways is inevitable. Moreover, although indigenous species play a crucial role in food and nutrition security, they cannot fully replace exotic ones. However, to combat malnutrition and mitigate the impacts of micronutrient deficiencies, every resource and strategy must be tapped to diversify diets and strengthen food and nutrition security. Specifically, indigenous food crops in the diet can reduce the double burden of malnutrition and improve dietary diversity in Rwanda. Hence, recommendations to revitalize the knowledge and consumption of indigenous crop and animal species within Rwanda include but are not limited to the following:

- Initiating comprehensive education about the importance of indigenous foods, both wild and cultivated. As older generations pass, the knowledge of indigenous species is lost, and younger generations cannot preserve them unless their importance is known. Therefore, integrating this aspect into school curricula would help future generations to value and conserve these precious riches.
- Investing in research about the nutritional value of African and Rwandan wild and domesticated indigenous foods. At present, research on the nutrition of indigenous foods in Rwanda is lacking. Illuminating the nutritional potential of these species will assist policymakers to create programs that will bolster their revival.
- Using existing development programs to encourage the use of indigenous food. For example, encouraging the cultivation of indigenous vegetables and fruits in the ongoing household kitchen garden program (*akarima k’igikoni*) would improve affordability of nutritious and diverse foods.

As Rwanda makes great strides in improving health, focusing on a more nutrition-sensitive agriculture has the potential to address micronutrient deficiency and malnutrition. Indigenous crops can combat these issues as a recent study noted, “…success in redressing the food, nutrition and health situation in the subcontinent hinges on revitalizing indigenous and traditional food systems, while at the same time promoting the use of biodiversity to ensure food availability and dietary diversity” [56].

## Data Availability

All data generated or analyzed during this study are included in this published article.
